# Sodium Intake, Dietary Knowledge, and Illness Perceptions of Controlled and Uncontrolled Rural Hypertensive Patients

**DOI:** 10.1155/2014/245480

**Published:** 2014-02-04

**Authors:** Aziz Kamran, Leila Azadbakht, Gholamreza Sharifirad, Behzad Mahaki, Afshan Sharghi

**Affiliations:** ^1^Public Health Department, School of Health, Ardabil University of Medical Sciences, Ardabil 5618953141, Iran; ^2^Health Education & Promotion Department, School of Health, Isfahan University of Medical Sciences, Isfahan 817473461, Iran; ^3^Food Security Research Center & Department of Community Nutrition, School of Nutrition and Food Science, Isfahan University of Medical Sciences, Isfahan, Iran; ^4^Department of Epidemiology and Biostatics, School of Public Health, Isfahan University of Medical Sciences, Isfahan, Iran; ^5^Community Medicine Department, School of Medicine, Ardabil University of Medical Sciences, Ardabil, Iran

## Abstract

*Introduction and Objectives*. Nutritional knowledge of the patients is important in dietary adherence. This study aimed to determine the relationship between illness perceptions and nutritional knowledge with the amounts of sodium intake among rural hypertensive patients. *Methods*. In a cross-sectional study, 671 hypertensive patients were selected in a multistage random sampling from the rural areas of Ardabil city, Iran, in 2013. Data were collected using a questionnaire consisting of four sections and were analyzed using Pearson correlation and multiple linear regressions by SPSS-18. *Results.* The mean of sodium intake in the uncontrolled hypertensive patients was 3599 ± 258 mg/day and significantly greater than controlled group (2654 ± 540 mg/day) (*P* < 0.001). Knowledge and illness perceptions could predict 47.2% of the variation in sodium intake of uncontrolled group. A significant negative relationship was found between knowledge and illness perceptions of uncontrolled hypertensive patients with dietary sodium intake (*r* = −0.66, *P* < 0.001 and *r* = −0.65, *P* < 0.001, resp.). *Conclusion.* Considering the fact that patients' nutritional knowledge and illness perceptions could highly predict their sodium intake, the importance of paying more attention to improve patients' information and perceptions about hypertension is undeniable, especially among the uncontrolled hypertensive patients.

## 1. Introduction

Excess sodium intake is associated with increased blood pressure [1]. High blood pressure is a major cause of cardiovascular disease worldwide, and recent studies showed that there is a linear correlation between blood pressure and cardiovascular disease [2]. In recent decades rapid social and economic changes have led to the increment in prevalence of cardiovascular risk factors such as blood pressure in Mediterranean and Middle Eastern countries. Additionally, according to what different studies report, the prevalence of hypertension has been reported to be 10 to 17% in these region countries [3]. Different results have been reported by several studies conducted in Iran. In general, it can be said that 25 to 35 percent of adults are affected by hypertension [4]. Because of its high prevalence, this disease is important; however, its importance has been doubled due to the fact that hypertension is an uncontrolled disease [5]. One of the most important factors which play a prominent role in controlling the disease is patient's adherence.

The World Health Organization defines adherence as the agreement between an individual's medication-related behaviors and following nutritional and lifestyle changes recommended by health care providers [6]. Adherence or orders and information compliance are influenced by patient's beliefs and their health conditions [7]. Some studies have shown negative attitudes of hypertensive patients toward their disease. These perceptions may also be important in patient compliance and performance improvement [8]. However, if the patient thinks that hypertension is a controllable disease, following the recommendations may be more likely [9].

Meanwhile, some studies have reported that trivializing the illness of patient is one of the reasons behind uncontrolled blood pressure [10, 11] and self-care is not satisfactory in these patients [12–14]. The role of nutrition in disease control is undeniable and diet is one of the most effective nonpharmacological strategies and studies evidenced that healthy nutrition has beneficial effects on cardiometabolic parameters [15–17], but behavior change and maintenance are not easy [18] because the greatest responsibility in dietary adherence is on the patients [19].

But, unfortunately, there are several conflicting evidences and most of the patients do not pay attention to dietary care instructions, only less than half of the patients have accepted healthy diet as part of their treatment [20], and studies have indicated wrong eating habits among them [21–23]. It seems that unhealthy behavior is rooted in misperceptions and incorrect knowledge of the nature of disease and its related nutritional issues, since poor knowledge of patients has been introduced as one of the reasons for the lack of blood pressure control [24, 25].

The main source of sodium intake in Iran is salt added at the table and in cooking, since processed foods in rural areas are not used, sodium added to these foods does not have an important role; therefore, knowledge of patients about the salt and its role in their illness can play a prominent role in dietary behavior. For this reason, the evaluation of knowledge related to hypertension in these patients is an integral part of overall health care activities [26] since knowledge has been introduced as an outcome of patients' education in interventional studies [27, 28]. The highest knowledge in these patients is associated with lower rates of quitting interventional programs and higher adherence to treatment and better control of the disease [26].

Therefore, considering the importance of nutrition in the management of hypertension and the key role of illness perceptions and knowledge in patient's adherence to nutrition and disease control, this study was conducted to determine the relationship between illness perceptions and salt knowledge with dietary sodium intake among rural hypertensive patients in the city of Ardabil in 2013.

## 2. Methods

This is a cross-sectional study conducted among 671 hypertensive patients who were referred to rural health care centers in Ardabil city, Iran, in 2013. Ardabil city is located in northwest of Iran, near Iran-Azerbaijan border. Two-stage random sampling method was used to achieve our total sample. Out of 13 health centers available in the rural area, 6 were selected during first stage randomly; then, in the second stage, participants from each center were chosen randomly from patients' records. After thoroughly explaining about the objectives of the study, we requested their willingness to participate in the study, and then after they agreed they were enrolled in the study. Men did not show more willingness to participate in the study and complete the questionnaire, whereas women were overrepresented.

The criteria for inclusion consisted of disease diagnosed by a physician and patient aged between 30 and 60 years without any complications of hypertension. Exclusion criteria were patients presenting with complications of hypertension, like stroke, cerebral vascular accidents (CVA), and so forth, and patients presenting with comorbidities like diabetes mellitus, renal failure, and ischemic heart disease (IHD). These exclusion criteria were considered because patients with other comorbid conditions might require special diets; therefore, the gathered data could not be generalized to hypertensive patients.

Systolic and diastolic blood pressures (BP) were measured with a qualified care provider in the rural health center after 15 minutes of rest, while the subject was in a seated and relaxed position. Two recordings were made at a 10-minute interval with millimeter mercury (mmHg) and the mean value of the 2 recordings (not varying by more than 5 mmHg) was calculated. The patients were classified as controlled hypertension (SBP < 140 mmHg and DBP < 90 mmHg) and uncontrolled hypertension (SBP ≥ 140 mmHg and DBP ≥ 90 mmHg) according to Seventh Report of the Joint National Committee on Prevention, Detection, Evaluation, and Treatment of High Blood Pressure (JNC 7) guidelines [29]. Body weight was measured with the participant wearing light clothing without shoes using a Seca weight scale to the nearest 0.1 kg and height was measured in centimeters (cm) using a stadiometer while the participant was standing in an upright position without wearing shoes. Body mass index (BMI) was calculated as weight in kg divided by height in meters squared. Patients having a BMI of less than 18.5 were classified as underweight, from 18.5 to less than 25 were considered normal, and from 25 to less than 30 were overweight, whereas those more than 30 were considered obese.

Data were collected by a four-part questionnaire that consisted of the demographic data such as gender, age, educational level, systolic and diastolic blood pressure, family history, and duration of illness and salt knowledge questions (each item was prepared as part of a standard answer: correct, incorrect, or do not know); the illness perception (14 questions with 4-point Likert option) and nutritional data were gathered by a 3-day food record questionnaire. Salt knowledge scores ranged between 20 and 0 and illness perception scores were ranged from 56 to 14.

The salt knowledge section included 20 questions about dietary recommendations on salt, salt intake and disease relationships, and the salt content of commonly eaten foods. All correct responses were scored as one, while incorrect responses which included “do not know” were assigned a score of zero.

Questionnaire was designed based on the literature review and face validity was approved by 5 experts in the field of health education in expert panel. Content validity was approved with content validity ratio (CVR) and content validity index (CVI) and reliability for nutritional knowledge section was 0.89 and for illness perception section was 0.92.

Illness perceptions consisted of a 14-item self-report scale that measured patient's cognitive and emotional representations of their illness including consequences, timeline, control, identity, and causes. Its validity was confirmed by an expert panel and the test demonstrates a good Pearson's test-retest correlation coefficient.

Each participant completed a 3-day food record, 2 weekdays and 1 weekend, to increase accuracy; first author provided 5 minutes of instructions to each participant on how to complete the food records. Also, participants were encouraged to consume usual amounts of typical food for the completion of the food record. Participants were asked to measure the volume of food consumed with household measurements (cups, tablespoons). After completing the food record, participants met the first author to review all the information for record accuracy and completeness and portion size of individual items on the food record. Nutritional data were analyzed by Nutritionist 4 software and sodium intake extracted.

Data were gathered by trained health professionals with interview method and SPSS version 18.0 for Windows (SPSS Inc., Chicago, IL, USA) was used for the data analysis. The demographic characteristics of the participants were reported by using descriptive statistics (frequencies, proportions, and means). The mean scores were compared with *t*-tests and one-way ANOVA and relationships were assessed by multiple linear regression and Pearsons correlation test.

## 3. Results

*Sociodemographic Characteristics of the Participants*.  Table 1 shows the sociodemographic characteristics of the participants. Out of a total of 671 patients, 74.8% were female, 433 patients were in the controlled group and 243 participants were uncontrolled. No significant difference was found in the two groups' family history, income, and educational levels. However, significant differences were seen in gender and BMI. Accordingly, compared with the controlled group, the mean of BMI was higher in the uncontrolled group and majority of uncontrolled group were men (Table 1).

In this study, sodium intake was significantly different based on age group between controlled and uncontrolled patients. The significant differences were also seen in the amount of sodium intake in all age groups and between the controlled and uncontrolled groups. Participants aged between 30 and 40 had received significantly more sodium in both groups and significant difference in sodium intake was found between the two groups considering level of education, income, and gender. But in the uncontrolled group, no significant difference was found in gender (male versus female) (*P* = 0.05) and income (*P* = 0.6) (Table 2).

Compared with the controlled group, the mean of sodium intake in the uncontrolled group was significantly higher. Controlled group significantly had higher mean scores in the knowledge and illness perception. In the present study, the mean of sodium intake in the uncontrolled group was 3599 ± 258 which was significantly greater than the controlled group (2654 ± 540) (*P* < 0.001) (Table 3).

Also, there was a significant negative correlation between sodium intake and knowledge and illness perception in both controlled and uncontrolled hypertensive patients while this correlation was greater among the uncontrolled patients (Table 4).

Using multiple linear regressions, the impact of the predictor variables on sodium intake was examined. The results of regression analysis were significant, after adjusting for energy intake, suggesting that the knowledge and illness perception variables accounted for (*R* ^2^) 13.6% of the variance in sodium intake in the controlled group and these variables predicted 47.2% of sodium intake in the uncontrolled patients. Other variables such as age, sex, disease duration, weight, and education were also entered into the model that did not have any significant relationship with the dependent variable (Table 5).

## 4. Discussion 

Hypertension progressively and permanently damages target organs, leading to life-threatening complications and death [30]. Considering the importance of nutrition's role in the hypertension control and the role of illness perception and nutritional knowledge in the nutritional adherence, to our knowledge, this is the first study which has examined the relationship between illness perception and knowledge with dietary sodium intake in rural hypertensive patients.

In the present study, no significant differences were seen between the two groups in demographic variables (age, income, educational level, and duration of disease) so it could be said that these two groups were similar in terms of demographic characteristics. In the present study, the mean score of illness perception significantly was higher in the controlled group. Perceptions and beliefs have an important role in the health and health behaviors [31, 32].

Some individuals did not have information about amount of needed salt per day [33]. In Grimes et al. (2009), 73% of participants were not informed of the maximum allowed amount of salt [34]. Lack of knowledge is one of the other barriers to reduce sodium intake in patients. In a qualitative study a patient said that “my doctor said that I do not add salt to food, and I have no other information" [35]. So compared with those with negative attitudes, people who have more knowledge are more likely to have healthy behavior and reduce their sodium intake [36].

In the present study, salt knowledge of the controlled patients was moderate while knowledge of the uncontrolled group was not acceptable. Considering the fact that in Iran patients take a continuous and free care by doctors and health workers in rural health centers, having a higher knowledge expectation of patients is not unreasonable. Our finding is consistent with similar studies conducted in Iran [30] and the study done by Oliveria et al. (2005) who obtained a similar result [37].

Despite this level of knowledge, patients were shown incorrect information about some aspects. In this study, for the question “hypertension dietary was only restricted for salt” about half of the patients gave the wrong answer. In the same study, the misinformation about the disease has been reported [37]. But in Chinese study, patient's knowledge was reported much lower and they had little information on nutrition [38].

In this study, based on education, income, and gender, no significant difference was found in sodium intake between the controlled and uncontrolled groups. These findings were consistent with other studies in this field and the differences between the feeding habits were attributed to demographic factors such as age, sex, education, and income [39].

Salt intake was attributed to demographic factors and those middle-aged male participants with lower income and lower education consumed more salt [1, 40]. However, in the present study, women received more sodium than men which is inconsistent with the results of previous studies such as Chung et al. (2004) [41] and Sheahan and Fields (2008) [35] in which the women consumed less salt.

These differences in results might be justified by the social and cultural paradox and nutritional pattern in Iranian rural areas and industrialized countries; however, further investigations are needed to find more comprehensive patterns of diet, especially in rural areas. But the importance of this study was explained by the fact that demographic factors could not be changed. Accordingly, only patient's perceptions and knowledge could be addressed with interventions. Sodium intake in the uncontrolled group was significantly higher, more than double the recommended amount [42, 43]; considering that salty taste in foods is desirable among Iranian people [33], this finding was expected.

But these patients are at high risk for complications of hypertension and it is very disturbing. However, despite that Iranian people are being cared for in the rural health care centers, the recommendations should be presented to them and high salt intake causes need a wider range of investigation. In a study conducted in Iran, adult women consumed 10 grams of salt per day which was 1.5 times more than the recommended daily amount [33]. No similar study on hypertensive patients was found in Iran, but the amount of sodium in American hypertensive patients was also 2.2 times more than the recommended dose [44].

The salty taste of food was stated as an important predictor of high salt consumption [45] and is considered as an important challenge for health workers. Excessive salt consumption in people who are accustomed to the salty taste is very difficult and requires a set of interventions. Meanwhile, the excess sodium intake is associated with increased blood pressure [1] and significantly increases the risk of heart attack [46, 47].

High blood pressure is a major factor in the etiology of cardiovascular disease [1]. Studies have demonstrated the importance of sodium in hypertension control repeatedly. For example, it is clear that reducing the sodium intake in patients and healthy subjects for 4 weeks or more, results in a significant reduction in blood pressure [48].

Pearson's correlation and linear regression showed negative significant relationships between knowledge and perceptions with sodium intake. In addition, the correlations were higher for both variables among the uncontrolled hypertensive group. Also, multiple linear regressions showed that knowledge and perception could predict 13.5% of the variation in sodium intake in controlled group while this value was 47.2% in uncontrolled group, respectively. It could be concluded that these two variables could play an important role in the feeding behaviors of the patient; this role was seen mainly among the uncontrolled group patients.

However, knowledge and perceptions play important roles in disease control and patient compliance; however, they do not guarantee patients' health behavior [32]. In addition to these variables, attention should be given to environmental factors such as health careers because blood pressure is influenced by many factors and this rate of prediction can be considered a very good prediction of the knowledge and perception. Vallés-Fernandez et al. (2009) in their study showed that blood pressure was uncontrollable, despite the good knowledge in patients. This was so because controlling BP was influenced by several factors including the patient-related factors (age, BMI, and lifestyle), factors associated with treatment, clinical evaluation factors (treatment techniques), and factors associated with medical equipment and facilities [49]. Also, in a similar study in Iran, despite good knowledge and attitudes of patients, blood pressure was not controlled [30]. Considering the same results, a detailed and comprehensive study about the causes of BP controlling is needed; it seems that in the current situation, all attention is paid to the patients and they will be recognized as original extradite but the fact is that patients have not participated in care designing and their participation is not considered. Evidences in some contexts indicated that the most important obstacle introduced to control blood pressure includes personnel's failure in implementing recommended health care [50].

This study had several limitations. First, as a cross-sectional study, the findings could only be used to examine associations and not to draw inferences regarding causality. Second, the model only explained about the variation in sodium intake. Therefore, future studies should be extended to study other factors which may mediate the relationship between socio-demographic factors and sodium intake such as self-efficacy, attitudes, and salt taste and finally, an overrepresentation of females occurred despite the use of random sampling methods which was another limitation in this study. This was due to greater compliance and a willingness among women to participate. In contrast, the multiple regression analysis, the optimal sample size, and the standard tools are the strengths of this study. The other strength, according to our knowledge, is that few studies have been done on the rural hypertensive patients even in urban patients in Iran and developing countries. In this study, dietary intake was assessed with 3 days of food recording instead of food recall, because 24 h food recall depended on memory and studies showed underreporting error with food recall tool [51, 52]; therefore, the results were more likely to be closer to reality.

## 5. Conclusions

Knowledge and illness perception play an important role in prediction of sodium intake variation in hypertensive patients. Therefore, the importance of paying more attention to these two variables in the design of interventions by health network planners and health professionals is essential. It seems necessary to explore the sources of patients' information and perceptions. Furthermore, trying to improve and update patients' information and providing a clear picture of the condition to influence their perceptions of their disease are also required.

## Figures and Tables

**Table 1:** Demographic characteristic of controlled^1^ and uncontrolled^2^ hypertensive patients.

Variable	Controlled (*N* = 433) Mean ± sd	Uncontrolled (*N* = 238) Mean ± sd	*P* value
Age	50.2 ± 6.6	50.9 ± 6.4	*P* = 0.6
Diseases duration	5.9 ± 4.0	5.5 ± 3.2	*P* = 0.2
BMI	29.2 ± 4.1	30.1 ± 5.0	*P* = 0.04
Systolic blood pressure	133.3 ± 7.5	153.5 ± 5.1	*P* < 0.001
Diastolic blood pressure	84.1 ± 5.4	91.1 ± 5.5	*P* < 0.001

	*N* (%)	*N* (%)	

Literature level			
Primary school	335 (77.4%)	173 (73.1%)	*P* = 0.3
Mid school	89 (20.6%)	58 (23.5%)	
High school	9 (2.1%)	8 (3.4%)	
Income			
Up to 4 million rial	192 (44.3%)	91 (38.2%)	*P* = 0.2
4–6 million rial	174 (40.2%)	110 (46.2%)	
More than 6 million rial	67 (15.5%)	37 (15.5%)	
Gender			
Male	95 (21.9%)	74 (31.1%)	*P* = 0.01
Female	338 (78.1%)	164 (68.9%)	
Family history			
Yes	215 (49.7%)	116 (48.7%)	*P* = 0.8
No	218 (50.3%)	122 (51.3%)	

^1^Controlled hypertensive patients: SBP < 140 mmHg and DBP < 90 mmHg.

^2^Uncontrolled hypertensive patients: SBP ≥ 140 mmHg and DBP ≥ 90 mmHg.

**Table 2:** Sodium intake among controlled^1^ and uncontrolled^2^ hypertensive patients.

Variables	Controlled (*N* = 433) Mean ± sd	Uncontrolled (*N* = 238) Mean ± sd	*P* value
Age			
30–40 years	2752 ± 462	3879 ± 436	*P* < 0.001
41–50 years	2562 ± 565	3575 ± 250	
51–60 years	2712 ± 521	3591 ± 222	
*P* value	*P* = 0.01	*P* < 0.001	
Literature level			
Primary school	2691 ± 545	3593 ± 230	*P* < 0.001
Mid school	2498 ± 510	3649 ± 545	
High school	2826 ± 389	3399 ± 69	
*P* value	*P* = 0.007	*P* = 0.03	
Income			
Up to 4 million rial	2562 ± 591	3598 ± 229	*P* = 0.001
4–6 million rial	2773 ± 493	3613 ± 274	
More than 6 million rial	2607 ± 446	3564 ± 279	
*P* value	*P* = 0.01	*P* = 0.6	
Gender			
Male	2473 ± 558	3552 ± 216	*P* < 0.001
Female	2705 ± 525	3621 ± 273	
*P* value	*P* < 0.001	*P* = 0.05	

^1^Controlled hypertensive patients: SBP < 140 mmHg and DBP < 90 mmHg.

^2^Uncontrolled hypertensive patients: SBP ≥ 140 mmHg and DBP ≥ 90 mmHg.

**Table 3:** Mean and standard deviation studied variables among controlled and uncontrolled hypertensive patients.

Variables	Controlled (*N* = 433) Mean ± sd	Uncontrolled (*N* = 238) Mean ± sd	*P* value
Dietary sodium intake (mg per day)	2654 ± 540	3599 ± 258	*P* < 0.001
Total energy intake (Kcl)	2420 ± 392	2755 ± 310	*P* < 0.001
Knowledge	15.1 ± 3.2	11.3 ± 4.5	*P* < 0.001
Illness perception	39.5 ± 9	32.1 ± 6.9	*P* < 0.001

**Table 4:** Correlation of knowledge and illness perception with sodium intake.

Variables	Pearson correlation	
	Correlation coefficient	*P* value
Controlled group (*N* = 433)		
Illness perception	−0.381	*P* < 0.001
Knowledge	−0.268	*P* < 0.001
Uncontrolled group (*N* = 238)		
Illness perception	−0.666	*P* < 0.001
Knowledge	−0.652	*P* < 0.001

**Table 5:** Multiple linear regressions of knowledge and illness perception in sodium intake variations (adjusted to energy intake).

Variables	Multiple linear regressions				*R* ^2^	Adjusted *R* square
	Beta	Std. error	*t*	*P* value		
Controlled group (*N* = 433)						
Model 1						
Illness perception	−0.232	2.4	−4.9	*P* < 0.001	0.136	0.132
Knowledge	−0.222	6.7	−4.7	*P* < 0.001		
Model 2	Model 1 + weight, age, diseases duration, gender				0.152	0.140
Uncontrolled group (*N* = 238)						
Model 1						
Illness perception	−0.362	3.6	−3.6	*P* < 0.001	0.472	0.467
Knowledge	−0.346	5.5	−3.4	*P* = 0.001		
Model 2	Model 1 + weight, age, diseases duration, gender				0.498	0.485
